# Treatment of bilateral congenital extensor tendon dislocation on multiple digits with only one sagittal band reconstruction: a case report

**DOI:** 10.3389/fped.2023.1078459

**Published:** 2023-06-23

**Authors:** Jun-Hyuk Lim, Sung-Taek Jung, Jae Hyeok Cheon, Sungmin Kim

**Affiliations:** Department of Orthopedic Surgery, Chonnam National University Medical School and Hospital, Gwangju, Republic of Korea

**Keywords:** congenital extensor tendon dislocation, multiple digits, sagittal band reconstruction, juncturae tendinum, surgical treatment

## Abstract

Bilateral congenital dislocation of the extensor tendon in the metacarpophalangeal joint is an exceedingly rare disease and often involves multiple fingers. Surgical treatment of multiple congenital extensor tendon dislocations in both hands has been reported; however, no report has clearly stated whether all fingers should be surgically treated in patients with multiple finger involvement. We report a case in which we successfully treated bilateral congenital extensor tendon dislocation on multiple digits with only one single-loop reconstruction of the sagittal band instead of operating on all involved fingers.

## 1. Introduction

Dislocation of the extensor tendons from their central position on the metacarpal heads can be traumatic, congenital, spontaneous, or degenerative (1–3). Congenital dislocation of the extensor tendons occurs mostly in children and affects multiple digits bilaterally (2, 3). The sagittal band that tightens the extensor hood and holds the tendons over the metacarpophalangeal (MCP) joints during flexion is the principal centralizing structure (1, 4). Other anatomic structures (i.e., intertendinous fascia and the juncturae tendinae) can behave as secondary stabilizers of the extensor tendons (3, 5). Failure of the sagittal bands to centralize the tendon and the absence of secondary stabilizers can cause dislocation of the extensor tendons.

Although various surgical techniques have been reported, the pathoanatomy of the dislocation and its appropriate treatment for congenital extensor tendon dislocation remains unknown due to the rarity of the condition. We report a case wherein we successfully treated bilateral congenital extensor tendon dislocation on multiple digits using a single-loop reconstruction of the sagittal band on a single digit.

## 2. Case description

**Figure 1:**
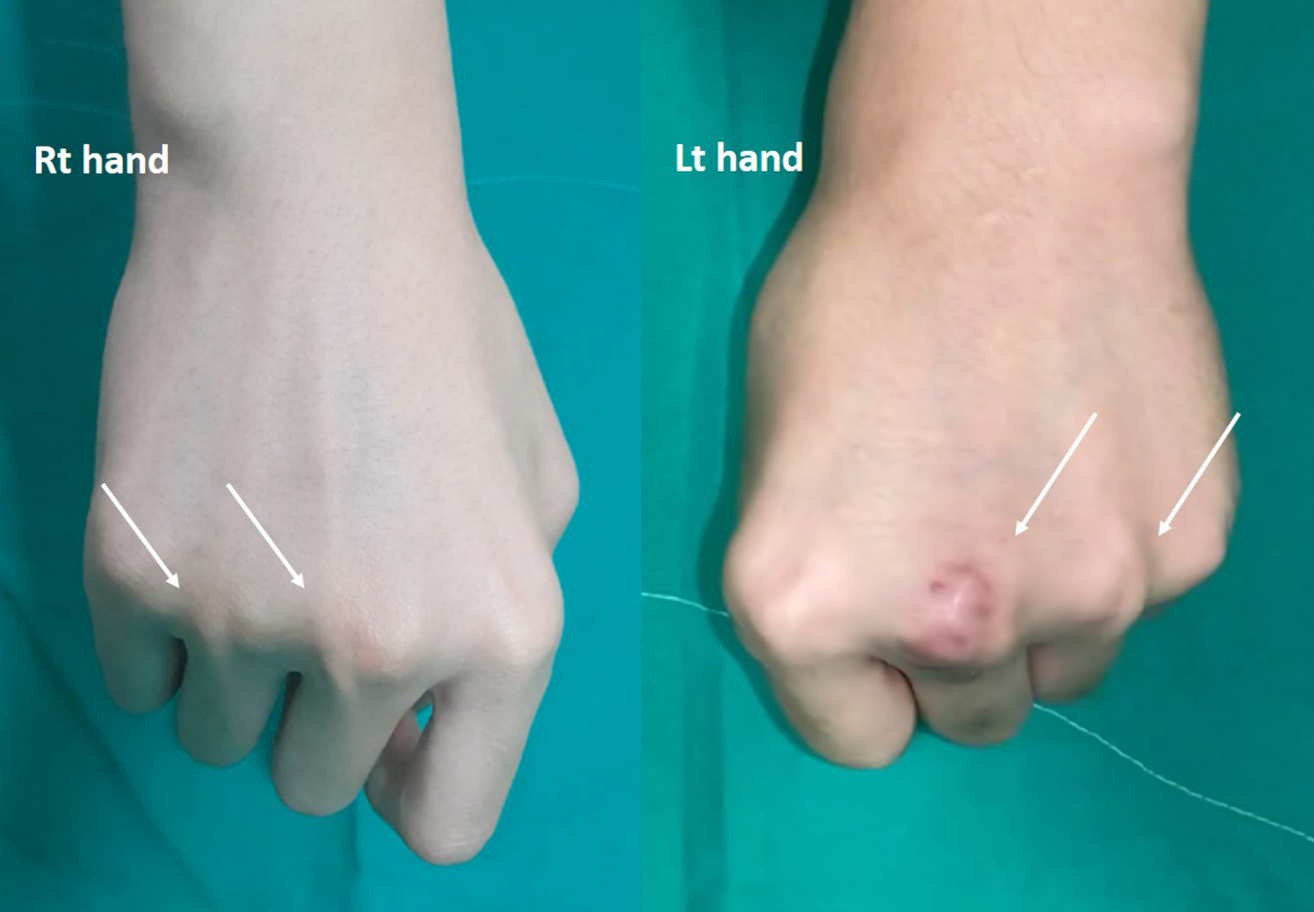
Ulnar side sagittal band dislocation of both third and fourth fingers.

This study is performed with an informed consent form signed by the patient and parents. A 14-year-old boy presented to our hospital with a complaint of pseudo-triggering accompanied by pain in the third and fourth fingers in both MCP joints bilaterally. The patient underwent a sagittal band repair on the left third finger after being diagnosed with traumatic sagittal band rupture at a local clinic 4 months prior. However, at the time of diagnosis, the patient had no history of trauma, and the dislocation presented in the third and fourth MCP joints on both hands. After the first surgical intervention, his parents noted that swelling, pain, and pseudo-triggering persisted. The patient also had noted the pseudo-triggering since childhood. A zigzag incisional wound on the left third MCP joint was visible on preoperative physical examination. With grasping, bilateral subluxation of extensor tendons of the third and fourth fingers on the metacarpal heads toward the ulnar side were observed (Figure 1). The Beighton score for generalized joint laxity was 3 points. We planned to perform a sagittal band reconstruction on the third and fourth finger with a 2-week interval starting with the unoperated right hand.

### 2.1. Surgical procedure and outcome

**Figure 2:**
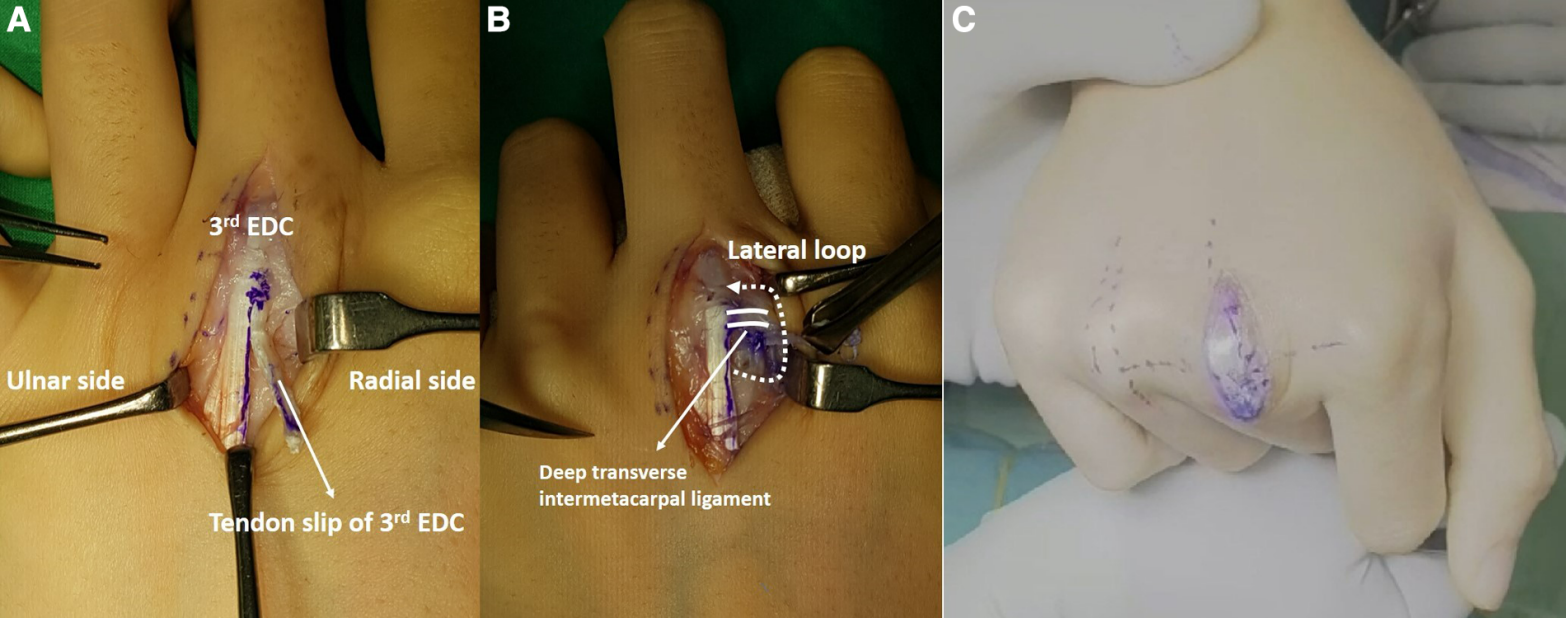
(**A,B**) Sagittal band reconstruction using tendon slip of the right third EDC using the Elson technique (6). The dotted line and arrow indicate the direction in which radial-side tendon slip of the third EDC passes under the deep transverse intermetacarpal ligament, forms a lateral loop, and returns to the third EDC tendon. The solid line indicates the deep transverse intermetacarpal ligament. (**C**) Disappeared sagittal band dislocation of right fourth finger after reconstruction of third sagittal band. EDC, extensor digitorum communis.

**Figure 3:**
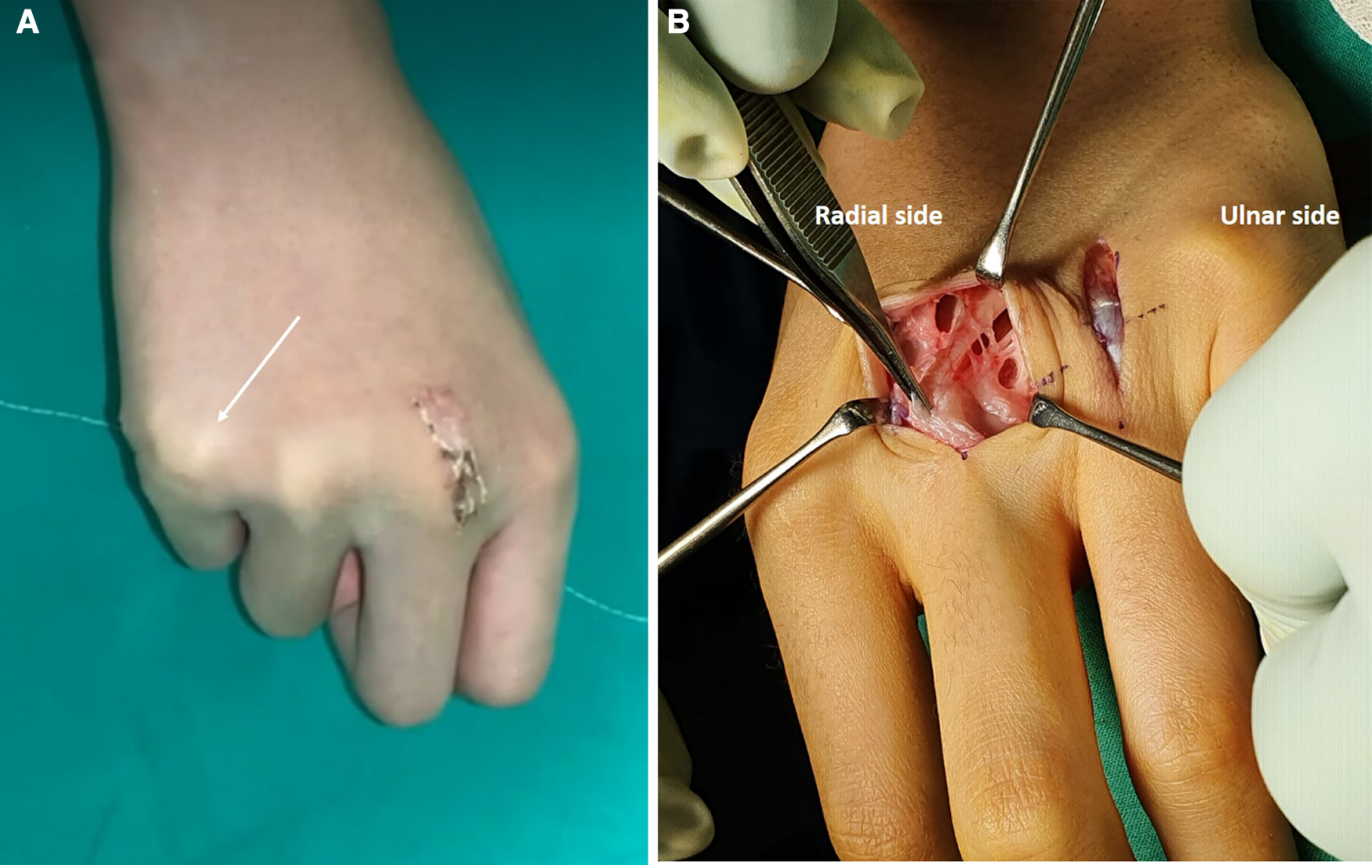
(**A**) A temporary radial-side dislocation of the right fifth extensor tendon after sagittal band reconstruction of the third finger. (**B**) Soft tissue adhesion on reconstruction site on the left third finger and no reconstruction of the fourth sagittal band.

A longitudinal, radial-side curved incision was made over the MCP joint on the right third finger. During surgery, we observed a defect on the radial sagittal band of the right third finger with no further obvious defects. The third sagittal band reconstruction was performed using the Elson technique (Figures 2A,B). After reconstruction of the right third sagittal band, we observed that the dislocation of the extensor tendon of the fourth finger was corrected spontaneously (Figure 2C). We decided to change the original surgical plan and avoid reconstruction of the sagittal band of the fourth finger and finished the right-hand operation. After 1 week from the operation, we observed a new radial-side dislocation of the extensor tendon of the right fifth finger (Figure 3A). We hypothesized that it was due to radial-side pulling force imposed from a reconstructed sagittal band. We followed-up the patient without any intervention. Radial-side extensor tendon dislocation of the right fifth finger was spontaneously resolved 5 days after the occurrence. After 2 weeks from the first operation on the right hand, we made a zigzag incision along the previous incision site on the left third finger and sagittal band reconstruction was performed using the same technique. Similarly, we decided not to reconstruct the left fourth sagittal band and finished the operation after confirming the spontaneous correction of the left fourth extensor tendon dislocation.

**Figure 4:**
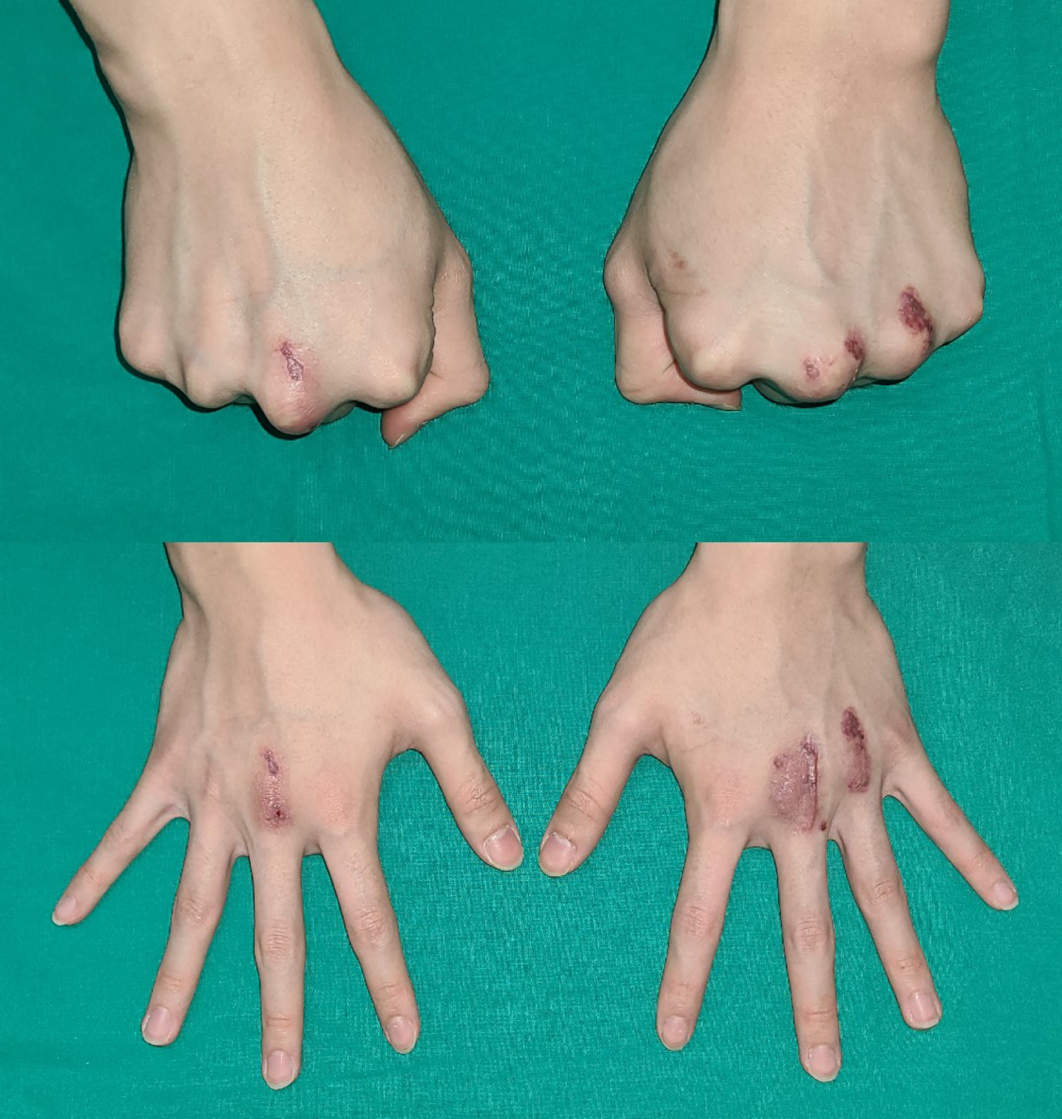
No recurrence of sagittal band dislocation and no pain after 18 months from operation.

Three months post-surgery, the ulnar side dislocation of the left third and fourth extensor tendons newly developed. We initially hypothesized that it was a recurred tendon dislocation and planned to have a revisional surgery on the third finger and a reconstruction on the fourth finger simultaneously. However, during the surgery, we observed adhesion of the surrounding soft tissue on the reconstruction site of the left third finger and did not find any evidence of failure on the reconstruction site. Accordingly, we carefully performed adhesiolysis and meticulously checked for dislocation of the left fourth extensor tendon. After the procedure on the third finger, the extensor tendon dislocation on the fourth finger was resolved spontaneously; hence, we could finish the surgery only with adhesiolysis without reconstruction of the fourth sagittal band (Figure 3B). The range of motion of the proximal interphalangeal joint was maintained with MCP joint immobilization in a 30° flexion under a short arm splint for 2 weeks after surgery. After 2 weeks, the splint was removed and passive joint motion of the MCP joint was performed. No recurrence of the extensor tendon dislocation or pain occurred in the MCP joint until 18 months post-surgery (Figure 4).

## 3. Discussion

Congenital sagittal band deficiency is a rare disease that occurs mostly in children. Although the related gene is not yet known, it is thought to be an autosomal dominant trait that may occur bilaterally and in multiple digits (3, 7–9). The main symptoms in patients with congenital extensor tendon dislocation include swelling around the MCP joint as well as pain and discomfort that worsen during flexion of the joint thus requiring surgical stabilization.

To date, various techniques have been reported for the surgical treatment of congenital extensor tendon reconstruction including soft tissue reconstruction using an extensor digitorum communis tendon slip (10) or a thin rectangular capsular flap (7) and indirectly increasing the tension of the sagittal band by repositioning the metacarpal bone without soft tissue surgery (11). Congenital extensor tendon dislocation is said to be caused by defects in juncturae tendinae, which is responsible for redistribution of the forces across the web and stabilization of the MCP joint (3, 12). A cadaveric study showed that juncturae tendinum and the sagittal band make an integrated contribution to MCP joint stabilization, especially during the coordinated digit flexion (13, 14). However, based on the intraoperative observation in the current study, it is unclear whether these two structures are the cause or if the intertendinous fascia and other structures responsible for recentering the extensor tendon may be the cause of dislocation (5).

This study reported the successful treatment of multiple congenital extensor tendon dislocation in a patient who underwent sagittal band reconstruction in only one digit in each hand. The dislocation of the fourth finger self-corrected after the reconstruction of the third finger on both sides. This finding suggested that there might be a connection between the extensor tendons and each tendon might be affected by pulling force. Therefore, the treatment of congenital multiple-digit extensor dislocation can be possible with only the most radial or ulnar side reconstruction without operating on all involved fingers. In addition, this hypothesis could explain the temporary subluxation of the fifth finger to the radial side. Further studies are needed to evaluate the connection between extensor tendons in patients with congenital extensor dislocation.

In our review of the literature, we found no reports on the direct repair of congenital extensor dislocation. Meanwhile, various sagittal band reconstruction techniques have been described in non-congenital extensor tendon dislocation cases including transposition of the extensor tendon with a displaced hood (15), creation of a retrograde slip with a part of the extensor tendon and rotating it beneath the deep intermetacarpal ligament (6) or around the volar interosseous muscle and anchoring it to the radial-side capsule (16), juncturae tendinum reconstruction (17), modification of the Elson technique using the extensor indicis proprius for the index finger sagittal band reconstruction in a patient with chronic sagittal band dislocation (18), and creation of a new pulley using a distended radial sagittal band and anchoring it to the surrounding extensor tendon (19). In our case, we observed that the dislocation recurred after a direct repair. As such, we believe that reconstruction surgery might be the better option compared to conservative or repair treatment due to a high possibility of the absence or deficiency of intertendinous structures, making it less likely to fail.

We found the self-resolution of the radial-side dislocation of the right fifth extensor tendon 5 days after sagittal band reconstruction of the third finger. We believe that self-resolution of the radial dislocation of the fifth extensor tendon may be due to stress relaxation. Stress relaxation is a mechanical property of tendons that refers to their ability to gradually decrease their tension or stress under constant deformation over time (20). In the case of radial-side dislocation of the fifth extensor tendon following sagittal band reconstruction of the third finger, the tension on fifth extensor tendon due to pulling force exerted on the juncturae tendinum may have initially increased due to the altered mechanics and forces in the hand. However, over time, the tension applied to the fifth extensor tendon may have undergone stress relaxation, leading to a decrease in tension and subsequent spontaneous improvement of the radial-side dislocation.

We found adhesions only on the third finger reconstruction site of the left hand and determined that this was due to pseudo-triggering recurrence. In addition, the left- and right-hand rehabilitation methods were slightly different from the method we initially presented to the patient. This was due to a delayed initiation of the range of motion (ROM) rehabilitation and the previous operation on the left finger. Many studies used buddy taping or application of a flexion-block splint for about 3–4 weeks post-surgery and performed active ROM to achieve full ROM for approximately 8 weeks. This recovery method has reportedly yielded positive results without dislocation recurrence or snapping (18, 19, 21). Therefore, active ROM after the removal of the splint is important for the prevention of adhesions.

## 4. Conclusions

In conclusion, surgical reconstruction may be required in symptomatic patients with multiple congenital extensor tendon dislocations. When multiple digits are involved, only reconstruction of the most radial or ulnar digit is necessary instead of operating on all involved fingers. In addition, proper active rehabilitation is essential to prevent postoperative adhesion.

## Data Availability

The original contributions presented in the study are included in the article, further inquiries can be directed to the corresponding author.
